# Supplementary material for: The leukocyte non-coding RNA landscape in critically ill patients with sepsis
Source: eLife. 2020 Dec 11;9:e58597. doi: 10.7554/eLife.58597 (PMC7775110; doi:10.7554/eLife.58597)
Supplement: Supplementary file 1. — Percentages depict the proportion of infections caused by the pathogen indicated. In total, 192 pathogens were assigned to 156 infections. In 40 (25.6%) infections, more than one pathogen was assigned as causative. [file elife-58597-supp1.docx]

**Supplementary File 1. Causative pathogens in critically ill patients with sepsis (n=156)**

| **Gram-positive bacteria** | | **63 (40.4%)** |  | **Gram-negative bacteria** | | **89 (57.1%)** |
| --- | --- | --- | --- | --- | --- | --- |
|  | *Streptococcus pneumoniae* | 22 (14.1%) |  |  | *Escherichia coli* | 20 (12.8%) |
|  | *Staphylococcus aureus* | 10 (6.4%) |  |  | *Pseudomonas aeruginosa* | 19 (12.2%) |
|  | *Enterococcus faecalis* | 6 (3.8%) |  |  | *Klebsiella pneumoniae* | 13 (8.3%) |
|  | *Streptococcus species* | 6 (3.8%) |  |  | *Haemophilus influenzae* | 11 (7.1%) |
|  | *Enterococcus faecium* | 5 (3.2%) |  |  | *Citrobacter species* | 5 (3.2%) |
|  | *Enterococcus species* | 4 (2.6%) |  |  | *Enterobacter cloacae* | 5 (3.2%) |
|  | *Streptococcus viridans* | 3 (1.9%) |  |  | *Bacteroides species* | 3 (1.9%) |
|  | *Clostridium species* | 1 (0.6%) |  |  | *Anaerobic bacteria* | 2 (1.3%) |
|  | *Mycoplasma pneumoniae* | 1 (0.6%) |  |  | *Enterobacteriaceae* | 2 (1.3%) |
|  | *Staphylococcus epidermidis* | 1 (0.6%) |  |  | *Klebsiella oxytoca* | 2 (1.3%) |
|  | *Streptococcus agalactiae* | 1 (0.6%) |  |  | *Acinetobacter baumannii* | 1 (0.6%) |
|  | *Streptococcus pyogenes* | 1 (0.6%) |  |  | *Legionella pneumophila* | 1 (0.6%) |
|  | Gram-positive bacilli (other) | 1 (0.6%) |  |  | *Morganella species* | 1 (0.6%) |
|  | Gram-positive cocci (other) | 1 (0.6%) |  |  | *Rickettsia rickettsii* | 1 (0.6%) |
|  |  |  |  |  | *Serratia marcescens* | 1 (0.6%) |
| **Viruses** | | **18 (11.5%)** |  |  | *Stenotrophomas maltophilia* | 1 (0.6%) |
|  | Influenza virus (incl. H1N1) | 10 (6.4%) |  |  | Gram-negative bacilli | 1 (0.6%) |
|  | Respiratory viruses | 5 (3.2%) |  |  |  |  |
|  | Cytomegalovirus | 1 (0.6%) |  | **Fungi** | | **12 (7.7%)** |
|  | Herpes simplex virus | 1 (0.6%) |  |  | Candida albicans | 5 (3.2%) |
|  | Other viruses | 1 (0.6%) |  |  | Aspergillus fumigatus | 3 (1.9%) |
|  |  |  |  |  | Candida species | 1 (0.6%) |
| **Other** | | **10 (6.4%)** |  |  | Candida glabrata | 1 (0.6%) |
|  | Other pathogens | 6 (3.8%) |  |  | Cryptococcus species | 1 (0.6%) |
|  | Polymicrobial or fecal flora | 4 (2.6%) |  |  | Pneumocystis jirovecii | 1 (0.6%) |
|  |  |  |  |  |  |  |
| **Unknown** | | **26 (16.7%)** |  |  |  |  |

Percentages depict the proportion of infections caused by the pathogen indicated. In total, 192 pathogens were assigned to 156 infections. In 40 (25.6%) infections, more than one pathogen was assigned as causative.
