# Supplementary material for: The leukocyte non-coding RNA landscape in critically ill patients with sepsis
Source: eLife. 2020 Dec 11;9:e58597. doi: 10.7554/eLife.58597 (PMC7775110; doi:10.7554/eLife.58597)
Supplement: Transparent reporting form [file elife-58597-transrepform.pdf]

eLIFE
1st Floor
24 Hills Road
Cambridge CB2 1JP, UK
P 01223 855340
W elifesciences.org
T @elife

eLife's transparent reporting form

We encourage authors to provide detailed information *within their submission* to facilitate the interpretation and replication of experiments. Authors can upload supporting documentation to indicate the use of appropriate reporting guidelines for health-related research (see EQUATOR Network), life science research (see the BioSharing Information Resource), or the ARRIVE guidelines for reporting work involving animal research. Where applicable, authors should refer to any relevant reporting standards documents in this form.

If you have any questions, please consult our Journal Policies and/or contact us: editorial@elifesciences.org.

Sample-size estimation

- You should state whether an appropriate sample size was computed when the study was being designed
- You should state the statistical method of sample size computation and any required assumptions
- If no explicit power analysis was used, you should describe how you decided what sample (replicate) size (number) to use

Please outline where this information can be found within the submission (e.g., sections or figure legends), or explain why this information doesn't apply to your submission:

The novelty of our study, that is, profiling non-coding RNA expression in leukocytes of patients with sepsis, precludes an adequate study power estimation. However, considering known co-regulation with protein-coding RNA expression, we provide study power estimates based on previous observations in typical gene expression studies (references: 8-10). Considering a false discovery rate of 5%, beta error level 5% (95% power), and typical effect sizes greater than 0.25 in sepsis relative to health, a sample size of 42 per group was estimated. In addition, 8 healthy volunteers in a human endotoxemia challenge would have more than 90% power to detect differences relative to pre-challenge (baseline) samples (references: 8, 10, 28-33). Using a continuous model, we estimated 156 patients would have more than 98% power to detect significant associations with demographic or clinical variables (false-discovery rates of 5%). This information is available in the methods section pertaining to "Microarrays and data processing".

Replicates

- You should report how often each experiment was performed
- You should include a definition of biological versus technical replication
- The data obtained should be provided and sufficient information should be provided to indicate the number of independent biological and/or technical replicates
- If you encountered any outliers, you should describe how these were handled
- Criteria for exclusion/inclusion of data should be clearly stated
- High-throughput sequence data should be uploaded before submission, with a private link for reviewers provided (these are available from both GEO and ArrayExpress)

Please outline where this information can be found within the submission (e.g., sections or figure legends), or explain why this information doesn't apply to your submission:

The above information is available in the methods sections pertaining to, "Patient population and inclusion criteria", "Healthy participants and human endotoxemia", "Microarrays and data processing", and "Availability of data and materials".

Statistical reporting

- Statistical analysis methods should be described and justified
- Raw data should be presented in figures whenever informative to do so (typically when N per group is less than 10)
- For each experiment, you should identify the statistical tests used, exact values of N, definitions of center, methods of multiple test correction, and dispersion and precision measures (e.g., mean, median, SD, SEM, confidence intervals; and, for the major substantive results, a measure of effect size (e.g., Pearson's r, Cohen's d)
- Report exact p-values wherever possible alongside the summary statistics and 95% confidence intervals. These should be reported for all key questions and not only when the p-value is less than 0.05.

Please outline where this information can be found within the submission (e.g., sections or figure legends), or explain why this information doesn't apply to your submission:

The above information is provided throughout the submission. Specifically, in the "Results" section (Figures 1-5 and legends), and "Materials and methods" section ("Microarrays and data processing", "Coexpression network and pathway analysis", and "Statistics")

(For large datasets, or papers with a very large number of statistical tests, you may upload a single table file with tests, Ns, etc., with reference to sections in the manuscript.)

Group allocation

- Indicate how samples were allocated into experimental groups (in the case of clinical studies, please specify allocation to treatment method); if randomization was used, please also state if restricted randomization was applied
- Indicate if masking was used during group allocation, data collection and/or data analysis

Please outline where this information can be found within the submission (e.g., sections or figure legends), or explain why this information doesn't apply to your submission:

Sample allocation is indictaed in the "Materials and methods" section, specifically, "Patient population and inclusion criteria", and "Healthy participants and human endotoxemia" sub-sections.
Masking was not used since our study focused on critically ill patients relative to healthy participants. The human endotoxemia trial was masked and information is provided in the "Materials and methods" section, "Healthy participants and human endotoxemia" sub-section.

Additional data files ("source data")

- We encourage you to upload relevant additional data files, such as numerical data that are represented as a graph in a figure, or as a summary table
- Where provided, these should be in the most useful format, and they can be uploaded as "Source data" files linked to a main figure or table
- Include model definition files including the full list of parameters used
- Include code used for data analysis (e.g., R, MatLab)
- Avoid stating that data files are "available upon request"

Please indicate the figures or tables for which source data files have been provided:

No "source data" files have been directly linked to main figures. All data used to derive graphs are available as normalized data matrices in GEO. The information is provided in the "Materials and methods" section, "Availability of data and materials" sub-section.

eLife Sciences Publications, Ltd is a limited liability non-profit non-stock corporation incorporated in the State of Delaware, USA, with company number 5030732, and is registered in the UK with company number FC030576 and branch number BR015634 at the address 1st Floor, 24 Hills Road, Cambridge CB2 1JP | August 2014
